# A Multicenter Cohort Study on DNA Methylation for Endometrial Cancer Detection in Cervical Scrapings

**DOI:** 10.1002/cam4.70361

**Published:** 2024-11-02

**Authors:** Xiao Ma, Xiaojun Chen, Jing Liang, Jingbo Zhang, Qixi Wu, Dong Wang, Xianghua Huang, Dan Zi, Dexin Chen, Hua Wan, Li Qu, Zhaoyun Jiang, Wenyu Shao, Jie Sun, Luyuan Chang, Yunchao Liu, Qin Zhang, Yanan Li, Yani Ding, Biao Tang, Fang Zhao, Hanqing Zhao, Dongyan Cao

**Affiliations:** ^1^ Department of Obstetrics and Gynecology, National Clinical Research Center for Obstetric & Gynecologic Diseases Peking Union Medical College Hospital, Chinese Academy of Medical Sciences, Peking Union Medical College Beijing China; ^2^ Department of Gynecologic Oncology Obstetrics and Gynecology Hospital of Fudan University Shanghai China; ^3^ Department of Obstetrics and Gynecology China‐Japan Friendship Hospital Beijing China; ^4^ Department of Medicine Beijing USCI Medical Laboratory Beijing China; ^5^ Department of Gynecologic Oncology Chongqing University Cancer Hospital Chongqing China; ^6^ Department of Gynecology Second Hospital of Hebei Medical University Shijiazhuang Hebei China; ^7^ Department of Gynecology Guizhou Provincial People's Hospital Guiyang Guizhou China; ^8^ Department of Gynecology Sichuan Provincial Maternity and Child Health Care Hospital Chengdu Sichuan China

**Keywords:** cervical scrapings, early detection, endometrial cancer, methylation, transvaginal ultrasonography

## Abstract

**Background:**

The increasing incidence of endometrial cancer (EC) has highlighted the need for improved early detection methods. This study aimed to develop and validate a novel DNA methylation classifier, EMPap, for EC detection using cervical scrapings.

**Methods:**

EMPap incorporated the methylation status of *BHLHE22* and *CDO1*, along with age and body mass index (BMI), into a logistic regression model to calculate the endometrial cancer methylation (EM) score for identifying EC in cervical scrapings. We enrolled 1297 patients with highly suspected EC, including 196 confirmed EC cases, and assessed the EMPap performance in detecting EC.

**Results:**

EMPap demonstrated robust diagnostic accuracy, with an area under the curve of 0.93, sensitivity of 90.3%, and specificity of 89.3%. It effectively detected EC across various disease stages, grades, and histological subtypes, and consistently performed well across patient demographics and symptoms. EMPap correctly identified 87.5% of the type II ECs and 53.8% of premalignant lesions. Notably, compared with transvaginal ultrasonography (TVS) in patients with postmenopausal bleeding, EMPap exhibited superior sensitivity (100% vs. 82.0%) and specificity (85.2% vs. 38.5%). In asymptomatic postmenopausal women, EMPap maintained high sensitivity (89.5%) and negative predictive value (NPV) (98.3%).

**Conclusions:**

This study demonstrated the potential of EMPap as an effective tool for EC detection. Despite the limited sample size, EMPap showed promise for identifying type II EC and detecting over 50% of premalignant lesions. As a DNA methylation classifier, EMPap can reduce unnecessary uterine interventions and improve diagnosis and outcomes.

## 1 Introduction

Endometrial cancer (EC) is a common gynecological malignancy originating from endometrium. It can be broadly categorized into two types: type I (endometrioid), affecting approximately 80% of patients, and type II (non‐endometrioid), affecting the remaining 20% [1].

Abnormal uterine bleeding (AUB) is a common symptom of EC. When a patient presents with AUB, especially postmenopausal bleeding (PMB), a biopsy during hysteroscopy or dilation and curettage (D&C) is usually recommended to detect or exclude EC. However, the risk of EC is observed in only 9% of women with PMB and 0.33% of premenopausal women with AUB [2, 3]. Therefore, many patients undergo unnecessary invasive procedures. Transvaginal ultrasonography (TVS) is commonly used to detect EC by measuring endometrial thickness (ET). However, its accuracy remains unsatisfactory, and the optimal threshold for positivity is debatable [4, 5, 6]. To address this, non‐invasive and accurate early warning detection for triaging patients for endometrial biopsy is essential. Such an approach would not only benefit triaging patients but also enhance EC screening.

DNA methylation, an epigenetic modification affecting tumor‐related genes, can serve as a biomarker for cancer detection [7, 8, 9]. Recent studies have identified several hypermethylated genes in ECs, including *ADCYAP1*, *BHLHE22*, *CDO1*, *PCDHGB7*, *ZNF454*, and *ZSCAN12* [10, 11, 12, 13]. These genes can be tested using non‐invasive methods, such as cervical scraping and tampons, enabling effective screening and triage of patients suspected of EC [10, 11, 12, 13, 14, 15]. Among these genes, *BHLHE22* and *CDO1* can be used to detect both type I EC and type II EC [16, 17]. Especially, their combined performance in detecting EC has good potential [17]. A multicenter study revealed that MPap, incorporating the methylation levels of *CDO1* and *BHLHE22* along with age and body mass index (BMI), effectively detects EC in women with AUB [18]. To further validate the capacity of *CDO1* and *BHLHE22* in identifying EC across different subtypes and subpopulations, our study introduces a classifier, EMPap. EMPap also combines the methylation levels of these two genes with age and BMI and utilizes a larger multicenter cohort for training and validation in women with suspected EC. Our investigation demonstrated the performance of EMPap in EC detection and assessed its efficacy in subpopulations with specific clinical characteristics. Additionally, we evaluated the impact of sampling timing on the accuracy of EMPap. Overall, our study provides compelling evidence for the clinical application of DNA methylation detection in the management of EC.

## 2 Materials and Methods

### 2.1 Subject Recruitment and Specimen Collection

From September 2021 to May 2023, we recruited women aged 18 or older with high suspicion of EC or newly diagnosed EC at seven hospitals in China. All enrolled patients underwent either endometrial biopsy or hysterectomy, and their pathological results were available. The inclusion criteria were as follows: (1) AUB or irregular vaginal discharge; (2) endometrial abnormalities detected by imaging (ET > 4 mm, endometrial heterogeneity, or polypoid masses) with or without symptoms; and (3) patients newly diagnosed with EC by endometrial biopsy where required to exclude EC. We excluded patients with pregnancy or puerperium, history of hysterectomy, relevant treatment for EC, or other gynecological malignancies.

The Ethics Committee of the Peking Union Medical College Hospital (No. ZS‐3064D), the ethics committees of the Obstetrics and Gynecology Hospital of Fudan University (No. 2021‐158), the Chongqing University Cancer Hospital (No. CZLS2022153‐A), the China‐Japan Friendship Hospital (No. AF‐BG‐06‐01.0), the Second Hospital of Hebei Medical University (No. 2022‐C016), the Guizhou Provincial People's Hospital (No. AF‐SOP‐035‐1.1), and the Sichuan Provincial Maternity and Child Health Care Hospital (No. 20230626‐192) approved the study.

**FIGURE 1:**
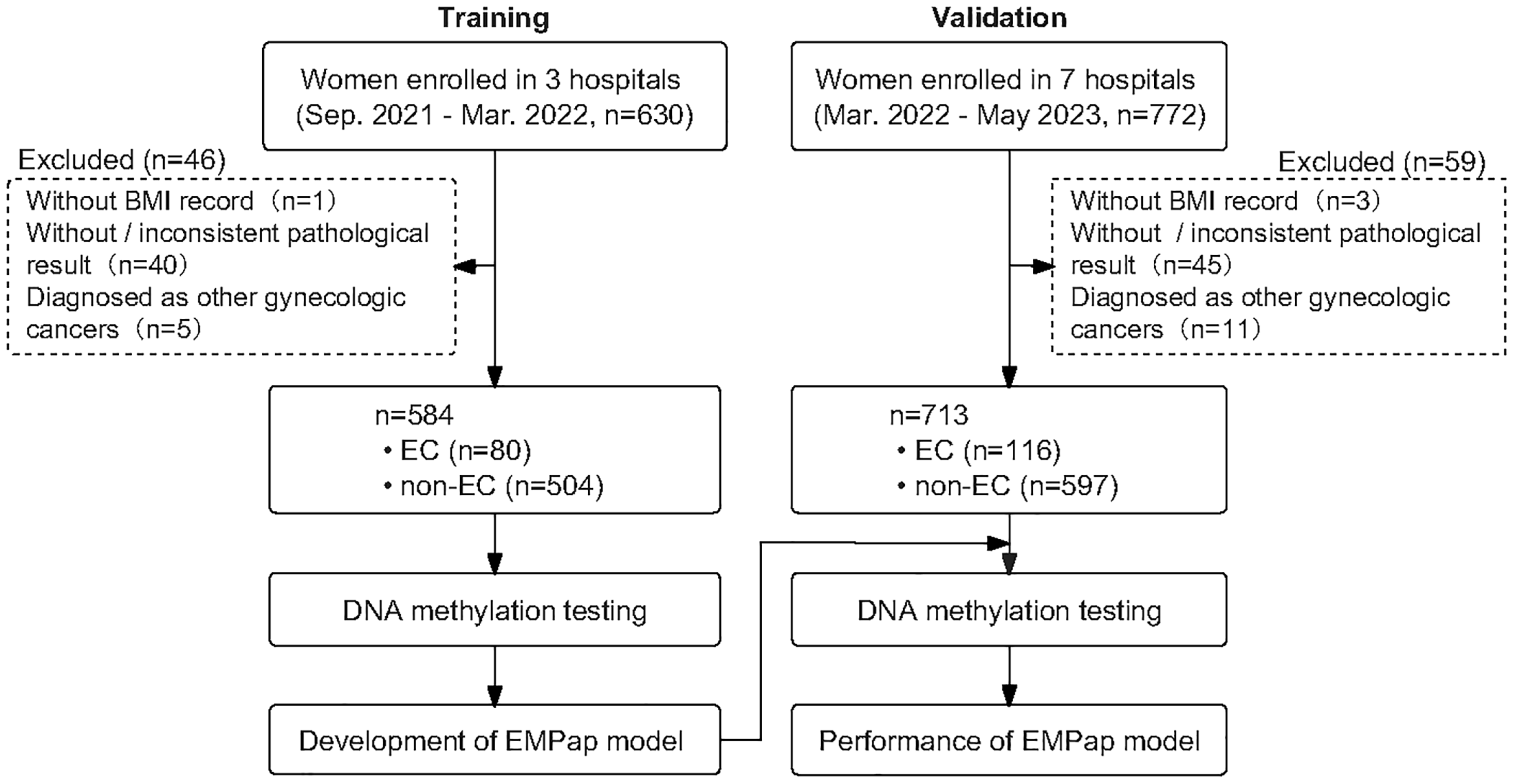
Schematic workflow of the development and validation of EMPap.

Each participant provided informed consent and underwent endometrial sampling before either an endometrial biopsy or hysterectomy. An experienced gynecologist collected the cervical scrapings by inserting a cervical brush (Cidabio, China) 2 cm deep into the cervical canal and rotating it twice. The brushes were stored at −20°C in collection tubes containing preservatives (Cidabio, China). The clinical information of the participants was also provided. Each specimen was independently assessed by at least two pathologists. Staging was performed according to the NCCN guidelines for uterine neoplasms, and surgical pathological staging was performed according to the International Federation of Gynecology and Obstetrics (FIGO) 2009 classification. Patients with inconsistent pathological results between the endometrial biopsy and hysterectomy specimens were excluded from the study, as we believe the final diagnosis should rely on the most significant pathological findings. Some patients with early or focal EC may have negative results in the hysterectomy despite positive initial biopsies, curettages, or hysteroscopies. Furthermore, since this is a multicenter study involving referral hospitals, some patients underwent a biopsy, curettage, or hysteroscopy and hysterectomy at different hospitals, making it challenging to obtain pathology slides from local hospitals for re‐evaluation. Therefore, we decided to exclude cases with discrepancies between biopsy and hysterectomy. We developed an EC detection model using samples from a training cohort and validated its performance using an independent cohort (Figure 1). Participants in the training cohort were recruited from the Peking Union Medical College Hospital, the Obstetrics and Gynecology Hospital of Fudan University, and the China–Japan Friendship Hospital between September 2021 and March 2022. An independent validation cohort included patients from three hospitals in the training cohort and four additional hospitals (Chongqing University Cancer Hospital, Second Hospital of Hebei Medical University, Guizhou Provincial People's Hospital, and Sichuan Provincial Maternity and Child Health Care Hospital) from March 2022 to May 2023.

### 2.2 DNA Methylation Assay

Genomic DNA was extracted from cervical scraping samples using the QIAamp DNA Mini Kit (QIAGEN, Germany) and subjected to bisulfite conversion using the EZ DNA Methylation‐Gold Kit (Zymo Research, USA). The methylation level of *BHLHE22* and *CDO1* was assessed using quantitative methylation‐specific PCR (qMSP). *COL2A1* served as the reference gene for quantification and quality control in the assay, as it is a CpG island‐free gene unaffected by methylation status [17]. Primer and probe sequences for *BHLHE22*, *CDO1*, and *COL2A1* were designed using Primer Express Software 3.0.1 (Applied Biosystems, USA). The targeted CpG sites and the sequences of primers and probes are shown in Table S1. The qMSP was conducted using TaqMan probe technologies and an ABI 7500 Real Time Fluorescence Quantitative PCR system (Life Tech, USA) with the following programs: activation at 95°C for 10 min, 50 cycles of denaturation at 95°C for 10 s, annealing and extension at 60°C for 40 s, and cooling at 40°C for 45 s. Each specimen was then subjected to qMSP. Trained technicians conducted the qMSP assays in the USCI laboratory.

### 2.3 Construction of the Classifier for Endometrial Cancer

Logistic regression and five‐fold cross‐validation were employed to construct a classifier model for detecting EC using the training samples. The clinical pathology results were used as reference standards. Based on the pathological findings, our cohort was divided into an EC group and a non‐EC group comprising normal endometrium (Normal), benign lesions (Benign), endometrial hyperplasia (EH), and atypical hyperplasia/endometrioid intraepithelial neoplasia (AH/EIN). We categorized the clinical pathology based on the highest grade of pathological findings. For example, cases of AH/EIN accompanied by EC were classified into the EC group. Relative quantification values (ΔCt) for *CDO1* and *BHLHE22* from qMSP, along with age and BMI, were used as independent predictors to construct the model. EC status (EC or non‐EC) was designated as a binary outcome variable. A logistic regression model was developed using the ‘glm’ package in R to examine the association between these variables and EC status. This logistic regression approach allowed us to determine the direction and strength of the relationship between each predictor and EC status, as represented by the respective regression coefficients. The model was evaluated on four of the five folds, reserving the fifth fold for the performance assessment. This process was repeated 10 times, yielding 50 distinct models (five‐fold multiplied by 10 repeats). The final model was derived by averaging the regression coefficients across all 50 models, and the score for each sample was obtained using the final model. In the training set, we determined the cutoff value for optimal differentiation between the EC and non‐EC groups. Subsequently, we assessed the efficacy of the classifier in identifying EC using a validation cohort. The study design is illustrated in Figure 1.

### 2.4 Statistical Methodologies and Analytical Framework

We tabulated the patient and tumor characteristics and evaluated the measurement data for normal distribution. When the data followed a normal distribution, we reported mean ± standard deviation (SD); otherwise, we used median (interquartile range, IQR). Categorical data are expressed as numbers (percentages), and continuous variables were compared using a two‐tailed Student's *t*‐test. Categorical clinical variables were analyzed using the chi‐squared test or Fisher's exact test and a detection model was created using logistic regression analysis. Significant differences in the model scores among the subgroups were analyzed using a nonparametric test (Kruskal–Wallis or Mann–Whitney *U* test). Propensity score matching was conducted to address bias related to high‐risk factors for EC by calculating the propensity score using logistic regression. The detection model's performance was visualized using receiver operating characteristic (ROC) curves and area under the curve (AUC) with corresponding 95% confidence intervals (CI). We determined the cutoff values of the classifier using Youden's index and calculated the sensitivity, specificity, positive predictive value (PPV), negative predictive value (NPV), and accuracy for the entire cohort and subgroups. All significant differences were assessed using two‐tailed *p* < 0.05. These analyses were conducted using R statistical package (version 4.2.3).

## 3 Results

### 3.1 Demographic and Clinical Profiles of the Studied Participants

In this study, 1402 women aged 18 or older with suspicion for EC were enrolled. Among them, 105 subjects were excluded: four owing to missing BMI information, 85 owing to insufficient or inconsistent pathologic findings, and 16 diagnosed with other gynecologic cancers. The remaining 1297 patients with confirmed pathology on endometrial biopsy or hysterectomy included 196 (15.1%) patients with EC, 39 (3.0%) with AH/EIN, 339 (26.1%) with EH, 227 (17.5%) with benign endometrial lesions, and 496 (38.2%) with normal endometrial tissue (Figure 1). A total of 196 EC cases included 18 (8.2%) AH/EIN cases accompanied by EC as we prioritized the most significant pathological findings in the diagnosis. Of 196 patients with EC, 16 did not undergo EC staging surgery before the end of the study, leaving their staging information unknown. The remaining 180 patients with EC completed standard staging surgery according to the 2009 FIGO criteria, and most of them were at stage I (85%). Specifically, 180 patients with EC were classified as having endometrioid adenocarcinoma (type I), whereas 16 were classified as having non‐endometrioid adenocarcinoma (type II), primarily comprising serous carcinoma, carcinosarcoma, and mixed carcinoma (Table S2).

In the patient population, the median age was 45 years and the median BMI was 23.2 kg/m^2^. Notably, the EC cohorts exhibited a statistically significant divergence from their non‐EC counterparts, with a higher median age and BMI (*p* < 0.001) as well as a greater prevalence of hypertension (*p* < 0.001 in the training cohort, *p* < 0.01 in the validation cohort) and diabetes (*p* < 0.001 in the training group, *p* < 0.05 in the validation cohort) than the non‐EC groups. Among the cohort, 63.1% reported AUB, a critical symptomatic indicator, with a pronounced occurrence in EC cases (86.7%) compared to non‐EC cases (58.86%). Table 1 summarizes the clinical characteristics of participants in the training and validation cohorts.

**TABLE 1:** Characteristics of patients.

Characteristic	Training cohort (*n* = 584)			Validation cohort (*n* = 713)		
	Non‐EC (*n* = 504)	EC (*n* = 80)	*p*	Non‐EC (*n* = 597)	EC (*n* = 116)	*p*
Age, year						
Median (IQR)	41 (15)	55 (15)	< 0.0001	45 (6)	58 (13)	< 0.001
BMI, kg/m^2^						
Median (IQR)	22 (4.2)	24.8 (5.5)	< 0.0001	23.4 (4.8)	25.1 (4.4)	< 0.001
Menopausal status						
Pre, *n* (%)	397 (78.8%)	32 (40.0%)	< 0.001	420 (70.4%)	28 (24.1%)	< 0.0001
Post, *n* (%)	107 (21.2%)	48 (60.0%)		177 (29.6%)	88 (75.9%)	
AUB						
Yes, *n* (%)	210 (41.7%)	66 (82.5%)	< 0.0001	438 (73.4%)	104 (89.7%)	0.0002
No, *n* (%)	294 (58.3%)	14 (17.5%)		159 (26.3%)	12 (10.3%)	
Hypertension						
Yes, *n* (%)	32 (6.3%)	22 (27.5%)	< 0.0001	77 (12.9%)	24 (20.7%)	0.009
No, *n* (%)	445 (88.3%)	54 (67.5%)		465 (77.9%)	89 (76.7%)	
Unknown, *n* (%)	27 (5.4%)	4 (5.0%)		55 (9.2%)	3 (2.6%)	
Diabetes						
Yes, *n* (%)	12 (2.4%)	11 (13.8%)	< 0.0001	25 (4.2%)	11 (9.5%)	0.0104
No, *n* (%)	465 (92.3%)	65 (81.3%)		518 (86.8%)	101 (87.1%)	
Unknown, *n* (%)	27 (5.4%)	4 (5.0%)		54 (9.0%)	4 (3.4%)	

Abbreviations: AUB, abnormal uterine bleeding; EC, endometrial cancer; the non‐EC including normal, benign lesions (such as polyp, myoma), benign endometrial hyperplasia, and endometrial atypical hyperplasia/endometrioid intraepithelial neoplasia (AH/EIN).

### 3.2 Establishment of EMPap in Detecting Endometrial Cancer

**FIGURE 2:**
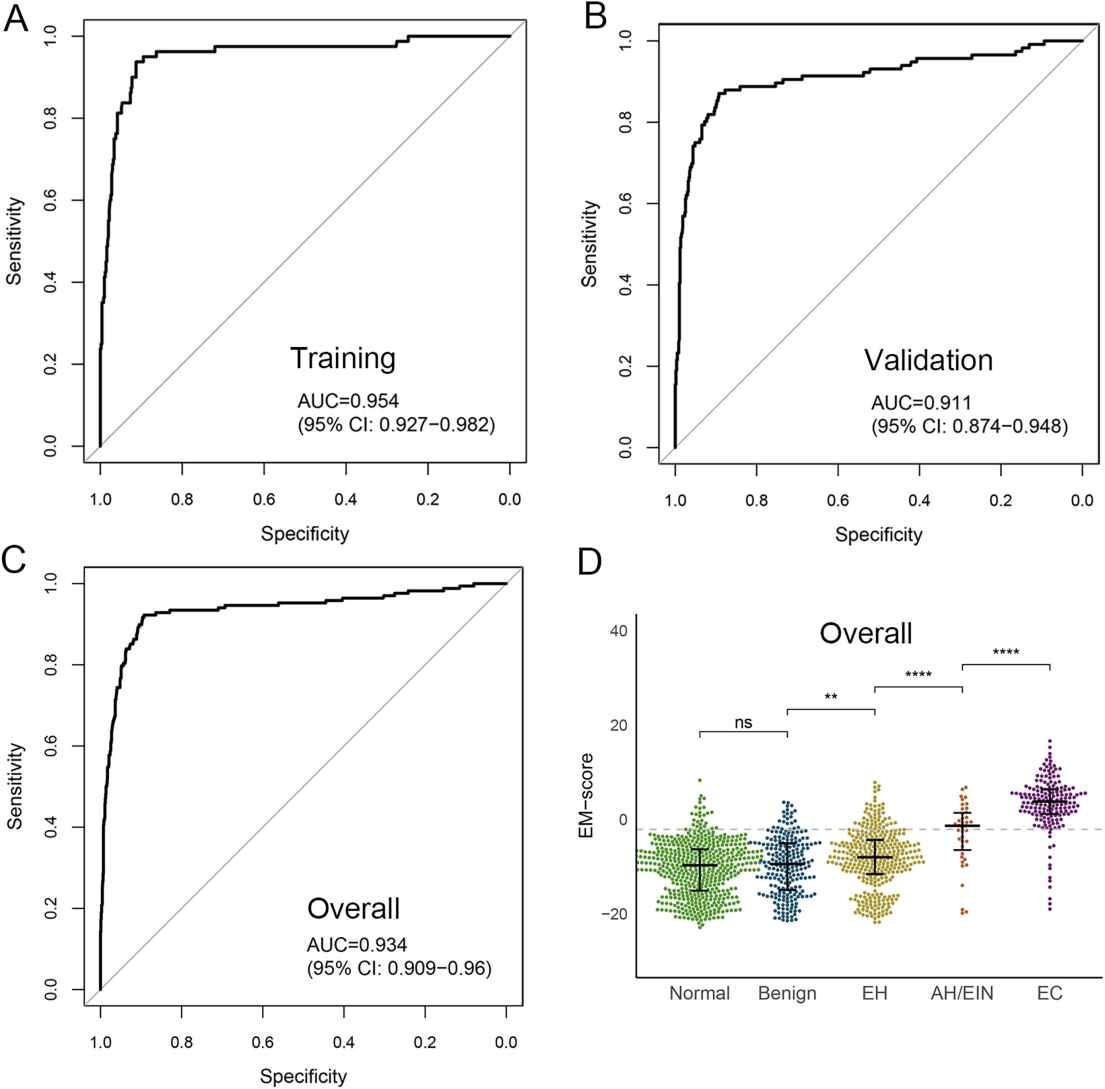
Area under the receiver operating characteristic curve (AUC) for EMPap in the training (A) and validation (B), and overall (C) cohorts and EMPap score (EM score) in the five pathology groups in the overall cohorts (D). EC, endometrial cancer; AH/EIN, atypical endometrial hyperplasia/endometrioid intraepithelial neoplasia; EH, endometrial hyperplasia without atypia; Benign, benign endometrial lesions such as polyps and myoma; Normal, normal physiological change. ns, no statistical difference; **, *p* < 0.05; ****, *p* < 0.001.

Methylation of *BHLHE22* and *CDO1* can predict the presence of EC in cervical scrapings, encompassing both type I and type II EC. Based on these findings, we developed a non‐invasive classifier, EMPap, for EC detection. EMPap integrated the methylation levels of *BHLHE22* and *CDO1* measured via qMSP along with age and BMI using a logistic regression model to calculate the endometrial cancer methylation (EM) score for EC identification. An optimal cutoff of −2.1 for the EM score was established based on the Youden index in the training cohort. Patients with an EM score above this threshold were considered to be at high risk for EC, whereas those below this threshold were considered to be at low risk. EMPap demonstrated good performance, distinguishing EC from non‐EC with a sensitivity of 93.8% and a specificity of 91.1% (Table 2), achieving an AUC of 0.95 (Figure 2A). Validation in an independent cohort confirmed EMPap's effectiveness, with a sensitivity of 87.9%, specificity of 87.8%, and AUC of 0.91 (Table 2, Figure 2B). Overall, EMPap proficiently differentiated EC from non‐EC cases in the overall cohort, achieving an AUC of 0.93, sensitivity of 90.3%, and specificity of 89.3% (Figure 2C, Table 2). The exceptional performance of EMPap reveals its potential as a good tool to detect EC non‐invasively.

**TABLE 2:** Performance of EMPap in detecting endometrial cancer.

Characteristics	Training cohort		Validation cohort		Overall cohort	
	Original	Adjusted^a^	Original	Adjusted^a^	Original	Adjusted^a^
ECs, *n*	80	78	116	90	196	168
Non‐ECs, *n*	504	504	597	597	1101	1101
Sensitivity, %	93.8	93.6	87.9	91.1	90.3	92.3
(95% CI)	(86.0–97.9)	(85.0–97.6)	(80.6–93.2)	(82.8–95.8)	(85.3–94.1)	(86.9–95.6)
Specificity, %	91.1	91.1	87.8	87.8	89.3	89.3
(95% CI)	(88.2–93.3)	(88.2–93.3)	(84.9–90.3)	(84.8–90.3)	(87.3–91.0)	(87.3–91.0)
PPV, %	62.5	61.9	58.3	52.9	60.0	56.8
(95% CI)	(53.2–71.0)	(86.0–97.9)	(50.6–65.6)	(44.8–60.9)	(54.1–65.6)	(50.7–62.7)
NPV, %	98.9	98.9	97.4	98.5	98.1	98.7
(95% CI)	(97.4–99.6)	(97.4–99.6)	(95.6–98.5)	(96.9–99.3)	(97.1–98.8)	(97.7–99.3)
Accuracy, %	91.4	91.4	87.8	88.2	89.4	89.7
(95% CI)	(89.0–93.1)	(89.0–93.1)	(85.0–90.7)	(85.6–90.8)	(87.5–92.0)	(87.7–91.6)

Abbreviations: EC, endometrial cancer; NPV, negative predictive value; PPV, positive predictive value; CI, confidence interval.

^a^ Excluding scraping samples collected within 10 days after biopsy.

### 3.3 Impact of Cervical Scraping Timing on EMPap Accuracy

The target of EMPap detection was the EC DNA shed from the uterine cavity into the cervix. To assess whether the efficacy of EMPap was affected by the timing of intrauterine operations such as biopsy sampling, we evaluated the correlation between the EC detection rate of EMPap and the timing of sampling before or after biopsy. We found 26 samples collected within 10 days after endometrial biopsy in the validation cohort and two samples collected in the training cohort. Excluding these patients resulted in a higher accuracy of EMPap, reaching 91.1% in the validation cohort and 92.3% in the overall cohort (Table 2). This finding suggests that the intrauterine procedure may influence the detection accuracy of EMPap.

To further analyze the correlation between detection accuracy and the timing of cervical scrapings, we conducted a stratification analysis of samples collected after biopsy in the validation cohort. The results revealed that the EC detection rate in the validation cohort was significantly lower for samples collected 1–10 days after biopsy than for those collected before or > 10 days after biopsy (Table S3).

These findings indicate that the timing of cervical scrapings affects the accuracy of EMPap for EC detection. Collection of cervical scrapings before biopsy yielded more accurate results.

### 3.4 EMPap Capability to Detect Endometrial Lesions across Various Subtypes

In our overall cohort, we conducted a stratified analysis of the histological subtypes of EC. The results, as shown in Table 3, revealed that EMPap correctly identified 90.6% (163/180) of type I ECs and 87.5% (14/16) of type II ECs. EMPap also demonstrated proficiency in detecting EC across various stages, with detection rates of 90.8% (139/153), 91.7% (11/12), 91.7% (11/12), and 66.7% (2/3) in the patients with stage I, II, III, and IV ECs, respectively. EMPap exhibited an excellent detection record for clear cell carcinomas, carcinosarcomas, mixed carcinomas, and undifferentiated carcinomas, except for one serous carcinoma and one mesonephric‐like adenocarcinoma. Furthermore, the capability of EMPap to correctly identify the grade of type I EC was equally impressive with success rates of 91.3% (63/69), 90.9% (70/77), and 88.0% (22/25) for patients with grades I, II, and III, respectively. These results showed that EMPap had a stable detection rate across all histological subtypes of EC.

**TABLE 3:** Detection rates of EMPap in histological subtypes of endometrial cancer.

Characteristic	Number	EMPap positive	Detection rate, % (95%CI)
Histological subtype (*N* = 196)			
Type I	180	163	90.6 (85.3–94.4)
Type II	16	14	87.5 (61.7–98.4)
Stage^a^ (*N* = 180)			
I	153	139	90.8 (84.8–94.7)
II	12	11	91.7 (61.5–99.8)
III	12	11	91.7 (61.5–99.8)
IV	3	2	66.7 (12.5–98.2)
Differentiation^b^ ^,^ ^c^ (*N* = 171)			
Grade I	69	63	91.3 (82.0–96.7)
Grade II	77	70	90.9 (82.2–96.3)
Grade III	25	22	88.0 (68.8–97.5)

^a^ The pathological stage of 16 samples was unknown. The stage classification was for the rest 180 ECs with records.

^b^ Differentiation classification was specific to the endometrioid carcinoma.

^c^ Nine patients had no differentiation record. The differentiation classification was for the rest of the 171 endometrioid carcinomas with records.

In addition, EMPap successfully detected focal EC in 66.7% (12/18) of AH/EIN cases. We also investigated the correlation between EM scores and histopathological outcomes. Notably, EM scores progressively increased from EH to AH/EIN and EC, highlighting the capacity of EMPap to discern the continuum from benign to precancerous and malignant endometrial states (Figure 2D).

### 3.5 EMPap Performance in Subpopulations with Specific Clinical Characteristics

EC typically has a higher incidence in older, obese, postmenopausal, women, and those with AUB, hypertension, or diabetes. Our study rigorously evaluated the efficacy of EMPap in these subpopulations (Table S4). Among women aged ≥ 50 in our study, EMPap demonstrated a sensitivity of 93.2% and a specificity of 82.9%. For women with a BMI ≥ 24 kg/m^2^, EMPap exhibited a sensitivity of 91.9% and a specificity of 77.8%. Similarly, among the postmenopausal women, EMPap consistently performed well, with a sensitivity of 93.4% and a specificity of 83.1%. In the AUB population, EMPap showed a sensitivity of 90.6% and a specificity of 88.4%. Additionally, EMPap performed consistently well among women with hypertension or diabetes. Notably, we observed slightly lower sensitivity and slightly higher specificity in women with low‐risk factors (except diabetes factor) compared to their counterparts (Table S4).

To address potential bias arising from differences in these characteristics between the EC and non‐EC groups, propensity score matching was performed. This process resulted in 188 matched pairs of EC and non‐EC cases through optimal pair matching (Table S5). The balanced cohort achieved a sensitivity of 89.9% (95%CI: 84.7%–93.8%) and a specificity of 83.5% (95%CI: 77.4%–88.5%). The stratification analysis of the balanced cohort yielded results similar to those of the original cohort (Table S4 and Table S5).

These results highlight the consistent and excellent performance of EMPap across diverse patient profiles, affirming its broad applicability. The higher sensitivity in the high‐risk group and the higher specificity in the low‐risk group indicate the potential of EMPap for stratification screening in EC risk assessment.

### 3.6 Diagnostic Potential of EMPap Compared to TVS for Patients with PMB

TVS is the preferred method for evaluating endometrial lesions using ET as an indicator of EC risk and is suitable for the initial EC risk evaluation of PMB. To evaluate the potential of EMPap in diagnosing EC, we compared the performance of EMPap and TVS in detecting EC. In our study, 183 women with PMB, including 61 with ECs and 122 non‐ECs, had a record of ET detected using TVS. We utilized 183 patients with PMB to assess the performance of EMPap for EC detection compared with that of TVS.

**FIGURE 3:**
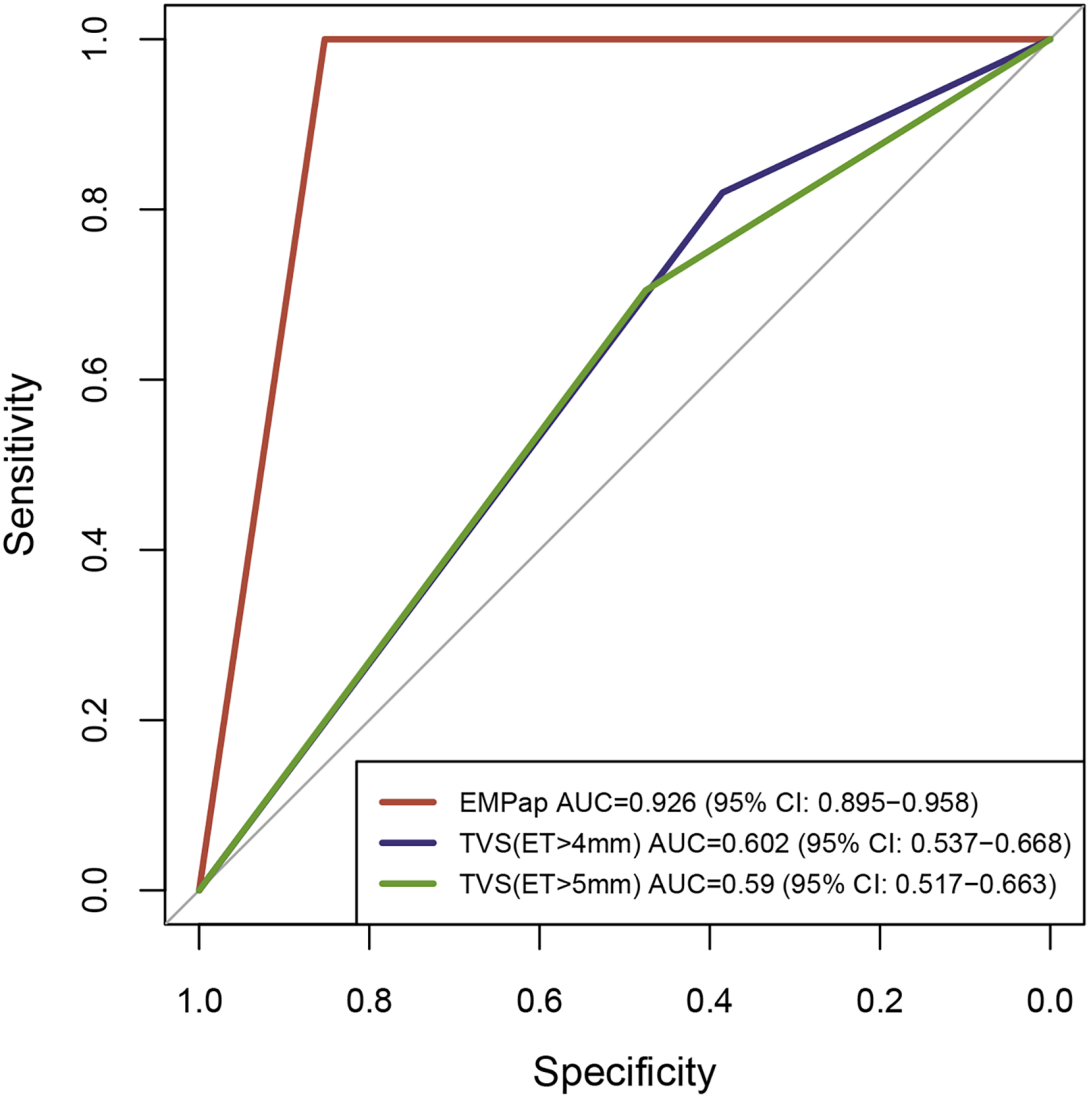
EMPap performance in comparison with TVS in women with PMB in the overall cohort. PMB, postmenopausal bleeding; TVS, transvaginal ultrasonography; ET, endometrial thickness.

In clinical guidelines, an ET > 4 mm or > 5 mm is a high‐risk indicator for EC. Among the 183 patients, 125 patients had ET > 4 mm, 40% had EC, 107 patients had ET > 5 mm, and 40.2% had EC. EMPap detected all 61 ECs, including 11 and 18 ECs missed by TVS at ET > 4 and > 5 mm, respectively. Specifically, for women with PMB and ET > 4 mm or > 5 mm, TVS had an AUC of 0.60 and 0.59, a sensitivity of 82.0% or 70.5%, and a specificity of 38.5% and 47.5%, respectively (Table S6, Figure 3). In contrast, the performance of EMPap was further underscored by its AUC of 0.93, sensitivity of 100.0%, and specificity of 85.2% in 183 women with PMB. These findings demonstrate the superiority of EMPap over TVS for detecting EC for PMB women with PMB.

Given that TVS may not adequately cover postmenopausal women without bleeding and premenopausal women, we evaluated the performance of EMPap in these subgroups (Table S7). Among the 162 postmenopausal women without bleeding, EMPap exhibited a sensitivity of 89.5%, a specificity of 79.7%, and a NPV of 98.3%. For the 877 premenopausal women, EMPap achieved a sensitivity of 83.3%, a specificity of 91.4%, and a NPV of 98.7%. These findings highlight the excellent exclusion capacity of EMPap, which effectively complements the population targeted by TVS.

## 4 Discussion

In this study, we developed the EMPap test to assess its significance in the early diagnosis of EC and evaluate its effectiveness in triaging patients with highly suspected EC who require further invasive endometrial biopsy. EMPap demonstrated impressive sensitivity (90.3%) and specificity (89.3%) for detecting EC. However, we observed that the EMPap performance was influenced by recent cervical scraping after biopsy. By excluding patients who had undergone a biopsy within 10 days before cervical scraping, EMPap sensitivity for detecting EC increased to 92.3%. Furthermore, EMPap consistently performed well across the different EC subtypes and specific patient characteristics. Notably, EMPap successfully identified 66.7% of patients with AH/EIN accompanied by EC, conditions that are often underestimated or misdiagnosed. For patients with PMB, EMPap outperformed TVS, with a significantly higher AUC, sensitivity, and specificity.

DNA methylation is a stable epigenetic marker that can be detected in cancerous tissues and various body fluids [19]. There is no significant difference in the diagnostic accuracy for EC between cytological samples and endometrial tissues [12]. Several studies have employed cervical swab sampling to explore DNA methylation as a screening tool for EC [10]. Compared with obtaining tumor tissues, this method has the following advantages: (1) cervical swabs can be sampled even if the patient presents with AUB (blood does not affect the assay) or when ultrasonography indicates endometrial lesions; (2) it is a less invasive or even non‐invasive procedure that can be conveniently conducted in an outpatient clinic; (3) this method benefits postmenopausal women with cervical atrophy and women who are not sexually active; and (4) with a short learning curve, it provides a convenient and efficient way to obtain specimens with high patient satisfaction.

We analyzed 1297 samples with highly suspected EC, including 196 EC and 39 AH/EIN samples. The proportions of EC and AH/EIN in our study exceeded those reported in previous studies. In our investigation, EMPap outperformed all previous similar studies [20]. It achieved an impressive AUC of 0.93, along with 90.3% sensitivity and 89.3% specificity for the entire cohort. EMPap accurately distinguished EC from non‐EC cases, not only for type I EC but also for type II EC. Among the 16 type II cases representing six different types, EMPap correctly identified 14 as high risk, suggesting promising prospects for non‐invasive screening of type II EC. In specific populations, EMPap consistently performed well in high‐risk patients with advanced age or obesity and those presenting with typical symptoms such as AUB or increased ET. These findings underscore the strong potential of EMPap as an efficient screening tool for detecting EC, transcending conventional symptomatic thresholds, and offering broader applicability for EC surveillance. Remarkably, AH and EC samples consistently yielded higher EM scores than EH samples, highlighting the ability of EMPap to reveal the gradual progression of endometrial lesions from benign to malignant. In summary, EMPap facilitates the clinical stratification of patients, aids in selecting those who require endometrial biopsy, and reduces the physical, mental, and economic burdens of unnecessary invasive examinations.

TVS is commonly used for the initial assessment of EC for patients with PMB when ET exceeds 4 mm [3, 15]. However, this study found that ET > 4 mm had low sensitivity (82.0%) and specificity (38.5%) for EC detection. In contrast, EMPap showed superior performance in identifying patients with PMB and thin ET, with or without symptoms. EMPap serves as a valuable complement to ultrasonography, particularly in the detection of type II ECs. Compared to TVS, EMPap has obvious advantages for EC screening diagnosis: approximately 90% sensitivity and specificity, independence from sampling time, and the menstrual cycle. It effectively stratified patients and accurately identified populations that required further invasive examinations for a definitive diagnosis. Unlike invasive procedures, such as D&C and hysteroscopy, EMPap is non‐invasive, causes less psychological trauma, and is cost‐effective. In addition, our study shows the importance of EMPap in premenopausal and postmenopausal women without bleeding, who are easily misdiagnosed with TVS. Overall, EMPap exhibited strong methodological feasibility and high performance compared to current clinical approaches for EC detection.

EC can be detected in pap smears, vaginal samples, and urine [14, 18, 21]. These methods demonstrate that shed endometrial tumor cells can be collected from the cervix, inspiring the development of novel methodologies to enhance EC detection rates. Our EMPap test, which utilizes cervical samples, has been proven to be effective for EC detection. Additionally, we investigated whether uterine procedures such as D&C or hysteroscopy, which may decrease malignant lesions within the uterine cavity or interfere with the cervical surface, could lead to false negatives. Analyzing the impact of biopsy sampling on EMPap results, we found that excluding patients who underwent biopsy within 10 days before cervical scraping increased sensitivity from 90.3% to 92.3% in the entire cohort. These findings suggest that intrauterine procedures may affect the concentration or availability of exfoliated endometrial cells and debris in the cervix. Our findings are consistent with a previous report by O'Flynn (2021), which advocated collecting cervical samples for EC detection before any clinical procedure or at least 2 weeks after a routine clinical diagnosis of EC [21]. Therefore, optimizing clinical procedures by collecting cervical samples before biopsy could enhance early detection and provide an effective triage to prevent unnecessary invasive procedures.

Given that most patients with EC exhibit AUB symptoms, several studies have used AUB populations to investigate methylation testing in EC [18, 22]. We compared the performance of our method in the AUB population to these studies. Our method achieved 90.6% sensitivity and 88.4% specificity in the AUB population. While sharing the same methylation markers and clinical variables with MPap, EMPap had a higher specificity (88.4% vs.71.5% ~ 73.8%) and a slightly lower sensitivity (90.6% vs. 92.5% ~ 92.9%) compared to MPap [18]. In comparison to the EPI‐SURE study [22], EMPap sensitivity in AUB women was similar to WID‐qEC (90.6% vs. 90.9%) but our specificity was lower (88.4% vs. 97.3%). In addition to the differences in biomarkers and methods, we observed that the EPI‐SURE obtained samples before any other clinical intervention. Therefore, we deduce that the difference in performance may be attributed to the fact that EPI‐SURE obtains samples before any clinical intervention, which is consistent with our findings on the impact of sampling timing on accuracy. Additionally, we compared our method with cytology for detecting EC. Despite the methodological differences, our method achieved similar results to urine and vaginal cytology (sensitivity 90.3% vs. 91.7%; specificity 89.3% vs. 88.8%) [21].

Owing to the economic situation in China and variations in medical conditions, facilities, and laboratory standards for molecular subtyping methods across the referral hospitals involved in this multicenter study, we initially did not include molecular subtyping in our study. However, recognizing its importance in the diagnosis and prognosis of EC, we will conduct further in‐depth research to enhance our understanding and improve existing detection methods.

## Author Contributions

**Xiao Ma:** conceptualization (equal), data curation (equal), formal analysis (equal), writing – original draft (equal), writing – review and editing (supporting). **Xiaojun Chen:** conceptualization (equal), data curation (equal), formal analysis (equal), writing – original draft (equal), writing – review and editing (supporting). **Jing Liang:** conceptualization (equal), data curation (equal), formal analysis (equal), writing – original draft (equal), writing – review and editing (supporting). **Jingbo Zhang:** conceptualization (equal), data curation (equal), formal analysis (equal), writing – original draft (equal), writing – review and editing (supporting). **Qixi Wu:** conceptualization (equal), data curation (equal), writing – review and editing (equal). **Dong Wang:** conceptualization (equal), investigation (equal), writing – review and editing (equal). **Xianghua Huang:** conceptualization (equal), investigation (equal), writing – review and editing (equal). **Dan Zi:** conceptualization (equal), investigation (equal), writing – review and editing (equal). **Dexin Chen:** conceptualization (equal), investigation (equal), writing – review and editing (equal). **Hua Wan:** data curation (equal), writing – review and editing (equal). **Li Qu:** data curation (equal), writing – review and editing (equal). **Zhaoyun Jiang:** data curation (equal), writing – review and editing (equal). **Wenyu Shao:** conceptualization (supporting), investigation (supporting), writing – review and editing (equal). **Jie Sun:** conceptualization (supporting), investigation (supporting), writing – review and editing (equal). **Luyuan Chang:** data curation (equal), writing – review and editing (supporting). **Yunchao Liu:** data curation (equal), writing – review and editing (supporting). **Qin Zhang:** conceptualization (supporting), investigation (supporting), writing – review and editing (supporting). **Yanan Li:** conceptualization (supporting), investigation (supporting), writing – review and editing (supporting). **Yani Ding:** conceptualization (supporting), investigation (supporting), writing – review and editing (supporting). **Biao Tang:** conceptualization (supporting), investigation (supporting), writing – review and editing (supporting). **Fang Zhao:** conceptualization (supporting), investigation (supporting), writing – review and editing (supporting). **Hanqing Zhao:** conceptualization (equal), data curation (equal), formal analysis (equal), investigation (equal), writing – original draft (lead), writing – review and editing (equal). **Dongyan Cao:** conceptualization (equal), data curation (equal), formal analysis (equal), investigation (equal), writing – original draft (lead), writing – review and editing (equal).

## Disclosure

The founder had no role in the study design, data collection and analysis, interpretation of the results, or the writing of the manuscript.

## Ethics Statement

This study was approved by the Ethics Committee of the Peking Union Medical College Hospital (No. ZS‐3064D). This study was approved by the ethics committees of the Obstetrics and Gynecology Hospital of Fudan University (No. 2021‐158), Chongqing University Cancer Hospital (No. CZLS2022153‐A), the China‐Japan Friendship Hospital (No. AF‐BG‐06‐01.0), the Second Hospital of Hebei Medical University (No. 2022‐C016), the Guizhou Provincial People's Hospital (No. AF‐SOP‐035‐1.1), and the Sichuan Provincial Maternity and Child Health Care Hospital (No. 20230626–192). Each participant signed an informed consent form before participating in the study.

## Conflicts of Interest

The authors declare no conflicts of interest.

## Supporting information


Data S1.

## Data Availability

The data generated in the present study may be requested from the corresponding authors.

## References

[1] J. V. Bokhman , “Two Pathogenetic Types of Endometrial Carcinoma,” Gynecologic Oncology 15 (1983): 10–17, 10.1016/0090-8258(83)90111-7. 6822361

[2] M. A. Clarke , B. J. Long , M. A. Del Mar , M. Arbyn , J. N. Bakkum‐Gamez , and N. Wentzensen , “Association of Endometrial Cancer Risk With Postmenopausal Bleeding in Women: A Systematic Review and Meta‐Analysis,” JAMA Internal Medicine 178 (2018): 1210–1222, 10.1001/jamainternmed.2018.2820. 30083701 PMC6142981

[3] M. E. Pennant , R. Mehta , P. Moody , et al., “Premenopausal Abnormal Uterine Bleeding and Risk of Endometrial Cancer,” BJOG: An International Journal of Obstetrics & Gynaecology 124 (2017): 404–411, 10.1111/1471-0528.14385. 27766759 PMC5297977

[4] L. Zhang , Y. Guo , G. Qian , T. Su , and H. Xu , “Value of Endometrial Thickness for the Detection of Endometrial Cancer and Atypical Hyperplasia in Asymptomatic Postmenopausal Women,” BMC Women's Health 22 (2022): 517, 10.1186/s12905-022-02089-y. 36510213 PMC9743752

[5] C. Saccardi , G. Spagnol , G. Bonaldo , M. Marchetti , R. Tozzi , and M. Noventa , “New Light on Endometrial Thickness as a Risk Factor of Cancer: What Do Clinicians Need to Know?,” Cancer Management and Research 14 (2022): 1331–1340, 10.2147/CMAR.S294074. 35401014 PMC8985823

[6] A. M. Cruz Garcia , E. Perez Morales , L. Ocon Padron , et al., “Asymptomatic Endometrial Thickening in Postmenopausal Women: Predictor of Malignant Pathology?,” Journal of Obstetrics and Gynaecology 43 (2023): 2160928, 10.1080/01443615.2022.2160928. 36576124

[7] M. Kulis and M. Esteller , “DNA Methylation and Cancer,” Advances in Genetics 70 (2010): 27–56, 10.1016/B978-0-12-380866-0.60002-2. 20920744

[8] E. Dulaimi , R. G. Uzzo , R. E. Greenberg , T. Al‐Saleem , and P. Cairns , “Detection of Bladder Cancer in Urine by a Tumor Suppressor Gene Hypermethylation Panel,” Clinical Cancer Research 10 (2004): 1887–1893, 10.1158/1078-0432.ccr-03-0127. 15041703

[9] S. C. Chang , P. L. Liew , M. Ansar , et al., “Hypermethylation and Decreased Expression of TMEM240 Are Potential Early‐Onset Biomarkers for Colorectal Cancer Detection, Poor Prognosis, and Early Recurrence Prediction,” Clinical Epigenetics 12 (2020): 67, 10.1186/s13148-020-00855-z. 32398064 PMC7218647

[10] R. V. den Helder , B. M. Wever , J. A. van Trommel , et al., “DNA Methylation Markers for Endometrial Cancer Detection in Minimally Invasive Samples: A Systematic Review,” Epigenomics 12 (2020): 1661–1672, 10.2217/epi-2020-0164. 32938224

[11] J. Yuan , Z. Mao , Q. Lu , et al., “Hypermethylated PCDHGB7 as a Biomarker for Early Detection of Endometrial Cancer in Endometrial Brush Samples and Cervical Scrapings,” Frontiers in Molecular Biosciences 8 (2021): 774215, 10.3389/fmolb.2021.774215. 35059435 PMC8763697

[12] L. Wang , L. Dong , J. Xu , et al., “Hypermethylated CDO1 and ZNF454 in Cytological Specimens as Screening Biomarkers for Endometrial Cancer,” Frontiers in Oncology 12 (2022): 714663, 10.3389/fonc.2022.714663. 35574348 PMC9095965

[13] C. Herzog , F. Marin , A. Jones , et al., “A Simple Cervicovaginal Epigenetic Test for Screening and Rapid Triage of Women With Suspected Endometrial Cancer: Validation in Several Cohort and Case/Control Sets,” Journal of Clinical Oncology 40 (2022): 3828–3838, 10.1200/JCO.22.00266. 36001862 PMC9671754

[14] B. M. M. Wever , R. van den Helder , A. P. van Splunter , et al., “DNA Methylation Testing for Endometrial Cancer Detection in Urine, Cervicovaginal Self‐Samples and Cervical Scrapes,” International Journal of Cancer 153 (2023): 341–351, 10.1002/ijc.34504. 36912267

[15] J. N. Bakkum‐Gamez , N. Wentzensen , M. J. Maurer , et al., “Detection of Endometrial Cancer via Molecular Analysis of DNA Collected With Vaginal Tampons,” Gynecologic Oncology 137 (2015): 14–22, 10.1016/j.ygyno.2015.01.552. 25677060 PMC4380654

[16] R. L. Huang , P. H. Su , Y. P. Liao , et al., “Integrated Epigenomics Analysis Reveals a DNA Methylation Panel for Endometrial Cancer Detection Using Cervical Scrapings,” Clinical Cancer Research 23 (2017): 263–272, 10.1158/1078-0432.CCR-16-0863. 27507616

[17] P. L. Liew , R. L. Huang , T. I. Wu , et al., “Combined Genetic Mutations and DNA‐Methylated Genes as Biomarkers for Endometrial Cancer Detection From Cervical Scrapings,” Clinical Epigenetics 11 (2019): 170, 10.1186/s13148-019-0765-3. 31779688 PMC6883641

[18] K. C. Wen , R. L. Huang , L. Y. Chen , et al., “Endometrial Cancer Detection Using a Cervical DNA Methylation Assay (MPap) in Women With Abnormal Uterine Bleeding: A Multicenter Hospital‐Based Validation Study,” Cancers (Basel) 14 (2022): 14, 10.3390/cancers14174343. PMC9454900 36077877

[19] J. Ibrahim , M. Peeters , G. Van Camp , and K. Op de Beeck , “Methylation Biomarkers for Early Cancer Detection and Diagnosis: Current and Future Perspectives,” European Journal of Cancer 178 (2023): 91–113, 10.1016/j.ejca.2022.10.015. 36427394

[20] J. E. Barrett , A. Jones , I. Evans , et al., “The WID‐EC Test for the Detection and Risk Prediction of Endometrial Cancer,” International Journal of Cancer 152 (2023): 1977–1988, 10.1002/ijc.34406. 36533702

[21] H. O'Flynn , N. A. J. Ryan , N. Narine , D. Shelton , D. Rana , and E. J. Crosbie , “Diagnostic Accuracy of Cytology for the Detection of Endometrial Cancer in Urine and Vaginal Samples,” Nature Communications 12 (2021): 952, 10.1038/s41467-021-21257-6. PMC7878864 33574259

[22] I. Evans , D. Reisel , A. Jones , et al., “Performance of the WID‐qEC Test Versus Sonography to Detect Uterine Cancers in Women With Abnormal Uterine Bleeding (EPI‐SURE): A Prospective, Consecutive Observational Cohort Study in the UK,” Lancet Oncology 24 (2023): 1375–1386, 10.1016/S1470-2045(23)00466-7. 37944542

