# Supplementary material for: A Multicenter Cohort Study on DNA Methylation for Endometrial Cancer Detection in Cervical Scrapings
Source: Cancer Med. 2024 Nov 2;13(21):e70361. doi: 10.1002/cam4.70361 (PMC11530713; doi:10.1002/cam4.70361)
Supplement: Supplementary file 1 — Data S1. [file CAM4-13-e70361-s001.docx]

# Supplementary Tables

**Table S1. Primers and Probes of qMSP in this study.**

| **Target** | **Sequence (5' - 3')** |
| --- | --- |
| ***BHLHE22*** |  |
| Forward | GAGATG**CG**G**CG**TTTAGT**CG**T |
| Reverse | A**CG**C**CG**AA**CG**C**CG**AATA |
| Probe | FAM-CTACAAC**CG**C**CG**AACTA-MGB |
| ***CDO1*** |  |
| Forward | **CG**TTTTTAG**CG**ATTT**CG**GATTTA |
| Reverse | ACCCAACATTAAAATAC**CG**AAA**CG** |
| Probe | FAM-TG**CG**A**CG**ATATTTT-MGB |
| ***COL2A1*** |  |
| Forward | GGGAAGATGGGATAGAAGGGAATAT |
| Reverse | AACAATTATAAACTCCAACCAC |
| Probe | VIC-TTCATTCTAACCCAATACCT-MGB |

Bold shows the CpG sites.

**Table S2.** **Pathological information of endometrial cancers in the entire cohort.**

| **Characteristics** | **Overall**  **(n=196)** |
| --- | --- |
| Histology |  |
| Type I | 180 (91.8%) |
| Endometrioid | 180 |
| Type II | 16 (8.2%) |
| Serous | 5 |
| Clear cell | 2 |
| Carcinosarcoma | 3 |
| MLA | 2 |
| Undifferentiated | 1 |
| Mixed | 3 |
| Stage |  |
| I | 153 (85%) |
| II | 12 (6.7%) |
| III | 12 (6.7%) |
| IV | 3 (1.7%) |
| Unknown | 16 |
| Differentiation |  |
| Grade I | 69 (40.4%) |
| Grade II | 77 (45.1%) |
| Grade III | 25 (14.6%) |
| Unknown | 9 |

MLA, Mesonephric-like adenocarcinoma

**Table S3. Detection rate of EMPap for EC at different scraping timing in the validation cohort.**

| **Scraping time** | **EC** | **EMPap high-risk** | **Detection rate** | ***p**** |
| --- | --- | --- | --- | --- |
| Before the biopsy | 41 | 39 | 95.1% | / |
| 1~10 days after the biopsy | 26 | 20 | 76.9% | **0.048** |
| 11~20 days after the biopsy | 18 | 16 | 88.9% | 0.5784 |
| 21~30 days after the biopsy | 10 | 9 | 90.0% | 0.4881 |
| Over 30 days after the biopsy | 18 | 15 | 83.3% | 0.1604 |
| NA | 3 | 3 | 100.0% | / |

*Fisher’s exact test was used to compare the EMPap detection rate between the scraping time before and after the biopsy.

EC, endometrial cancer

**Table S4. Sensitivity and specificity of EMPap in specific populations with different characteristics in the overall cohort.**

| **Subgroup** | **Sensitivity, % (95% CI)** | | **Specificity, % (95% CI)** |
| --- | --- | --- | --- |
| Age, year | |  |  |
| < 50 (n=804) | | 81.3 (66.9-90.6) | 92.2 (90.0-94.0) |
| ≥ 50 (n=493) | | 93.2 (87.6-96.5) | 82.9 (78.4-86.6) |
| BMI, kg/m^2^ | |  |  |
| < 24 (n=767) | | 87.5 (77.6-94.1) | 96.0 (94.2-97.3) |
| ≥ 24 (n=530) | | 91.9 (85.3-95.8) | 77.8 (73.4-81.7) |
| Menopause | |  |  |
| No (n=877) | | 83.3 (71.5-91.7) | 91.4 (89.3-93.3) |
| Yes (n=420) | | 93.4 (87.8-96.9) | 83.1 (78.2-87.3) |
| AUB | |  |  |
| No (n=479) | | 88.5 (69.8-97.6) | 90.5 (87.4-93.0) |
| Yes (n=818) | | 90.6 (85.2-94.5) | 88.4 (85.7-90.8) |
| Hypertension | |  |  |
| No (n=1053) | | 88.1 (86.2-90.1) | 90.3 (88.5-92.1) |
| Yes (n=155) | | 95.7 (92.4-98.9) | 79.8 (73.4-86.2) |
| Diabetes | |  |  |
| No (n=1149) | | 90.4 (88.7-92.1) | 89.6 (87.9-91.3) |
| Yes (n=59) | | 86.4 (77.4-95.3) | 73.0 (61.4-84.5) |

BMI, body mass index; AUB, abnormal uterine bleeding.

**Table S5. Propensity score matching analysis**

| **Characteristics** | **PSM cohort (n=376)** | | | | **Performance** | |
| --- | --- | --- | --- | --- | --- | --- |
|  | **non-EC** | | **EC** | ***p*** | **Sensitivity, % (95% CI)** | **Specificity, % (95% CI)** |
| Age, year  Median (IQR) |  |  | |  |  |  |
|  | 54 (10) | 57 (13) | | 0.33 |  |  |
| < 50 (n=91) | / | / | |  | 80.4 (66.1-90.6) | 86.7(73.2-94.9) |
| ≥ 50 (n=285) | / | / | |  | 93.0 (87.4-96.6) | 82.5 (75.3-88.4) |
| BMI, kg/m^2^ |  |  | |  |  |  |
| Median (IQR) | 25 (4.55) | 25 (5.13) | | 0.29 |  |  |
| < 24 (n=135) | / | / | |  | 86.6 (76.0-93.7) | 92.6 (83.7-97.6) |
| ≥ 24 (n=241) | / | / | |  | 91.7 (85.3-96.0) | 78.3 (69.9-85.3) |
| Menopause |  |  | |  |  |  |
| No (n=113) | 56 (29.8%) | 57 (30.3%) | | 0.91 | 82.5 (70.1-91.3) | 85.7 (73.8-93.6) |
| Yes (n=263) | 132 (70.2%) | 131 (69.7%) | |  | 93.1 (87.4-96.8) | 82.6 (75.0-88.6) |
| AUB |  |  | |  |  |  |
| No (n=45) | 22 (11.7%) | 23 (12.2%) | | 0.87 | 87.0 (66.4-97.2) | 77.3 (54.6-92.2) |
| Yes (n=331) | 166 (88.3%) | 165 (87.8%) | |  | 90.3 (84.7-94.4) | 84.3 (77.9-89.5) |
| Hypertension |  |  | |  |  |  |
| No (n=287) | 144 (76.6%) | 143 (76.1%) | | 0.90 | 88.1 (81.6-92.9) | 83.3 (76.2-89.0) |
| Yes (n=89) | 44 (23.4%) | 45 (23.9%) | |  | 95.6 (84.9-99.5) | 84.1 (69.9-93.4) |
| Diabetes, No. |  |  | |  |  |  |
| No (n=336) | 170 (90.4%) | 166 (88.3%) | | 0.50 | 90.4 (84.8-94.4) | 84.7 (78.4-89.8) |
| Yes (n=40) | 18 (9.6%) | 22 (11.7%) | |  | 86.4 (65.1-97.1) | 72.2 (46.5-90.3) |

BMI, body mass index; AUB, abnormal uterine bleeding; EC, endometrial cancer; the non-EC including normal, benign lesions (such as polyp, myoma), benign endometrial hyperplasia, and endometrial atypical hyperplasia/endometrioid intraepithelial neoplasia (AH/EIN).

**Table S6. The performance of EMPap compared with TVS in PMB women.**

| **Characteristics** | **EMPap** | **TVS (ET > 4 mm)** | **TVS (ET > 5 mm)** |
| --- | --- | --- | --- |
| High-risk, n | 61 | 50 | 43 |
| Low-risk, n | 18 | 75 | 64 |
| Sensitivity, % (95% CI) | 100 (94.1-100) | 82.0 (70.0-90.6) | 70.5 (57.4-81.5) |
| Specificity, % (95% CI) | 85.2 (77.7-91.0) | 38.5 (29.9-47.8) | 47.5 (38.4-56.8) |
| PPV, % (95% CI) | 77.2 (65.1-85.6) | 40.0 (31.5-49.2) | 40.2 (31.0-50.1) |
| NPV, % (95% CI) | 100 (95.6-100.0) | 81.0 (68.2-89.7) | 76.3 (64.9-85.0) |
| Accuracy, % (95% CI) | 90.2 (84.9-94.1) | 53.0 (45.5-60.4) | 55.2 (47.7-62.5) |

TVS, transvaginal ultrasonography; PMB, postmenopausal bleeding; EC, endometrial cancer; ET, endometrial thickness; PPV, positive predictive value; NPV, negative predictive value.

**Table S7. EMPap performance in postmenopausal women without bleeding and premenopausal women.**

| **Characteristics** | **EMPap in postmenopausal women without bleeding** | **EMPap in premenopausal women** |
| --- | --- | --- |
| Sensitivity, % (95% CI) | 89.5 (66.9-98.7) | 83.3 (71.5-91.7) |
| Specificity, % (95% CI) | 79.7 (72.2-86.0) | 91.4 (89.3-93.3) |
| PPV, % (95% CI) | 37.0 (23.2-52.5) | 41.7 (32.7-51.0) |
| NPV, % (95% CI) | 98.3 (93.9-99.8) | 98.7 (97.6-99.4) |

EC, endometrial cancer; ET, endometrial thickness; PPV, positive predictive value; NPV, negative predictive value.
